# P-1559. Comparing Patient and Clinician Perceptions of Health-Related Quality of Life in Hospitalized Patients with Complicated Urinary Tract Infections: A Qualitative Descriptive Study

**DOI:** 10.1093/ofid/ofae631.1726

**Published:** 2025-01-29

**Authors:** Jessica R Howard-Anderson, Mersedes Brown, Rachel E Korn, Julie Miller, Katherine Norman, Felicia Ruffin, Sherry Thomas, Engels N Obi, Nireesha Sidduri, Alexandre H Watanabe, Emre Yucel, Hayden Bosworth, Helen Boucher, Deborah Collyar, Sarah B Doernberg, Megan Oakes, Bryce B Reeve, Vance G Fowler, Heather King

**Affiliations:** Emory University, Atlanta, Georgia; Duke University School of Medicine, Durham, North Carolina; Duke University Medical Center, Durham, North Carolina; Duke University School of Medicine, Durham, North Carolina; Duke University, Durham, North Carolina; Duke University Medical Center, Durham, North Carolina; Duke University, Durham, North Carolina; Merck & Co., Inc, BASKING RIDGE, New Jersey; Merck, Philadelphia, Pennsylvania; Merck & Co., Inc., South Orange, New Jersey; Merck & Co., Inc., South Orange, New Jersey; Duke University, Durham, North Carolina; Tufts University School of Medicine, Boston, MA; Patient Advocates In Research (PAIR), Danville, California; University of California, San Francisco, San Francisco, CA; Duke University, Durham, North Carolina; Duke University School of Medici, Durham, North Carolina; Duke University Medical Center, Durham, North Carolina; Duke/Durham VA, Durham, North Carolina

## Abstract

**Background:**

There is limited research directly comparing patient and clinician-reported health-related quality of life (HRQoL) in bacterial infections. In this qualitative study, we compared patient and their treating clinician perceptions of HRQoL in complicated urinary tract infections (cUTI).

Table 1

**Figure:**
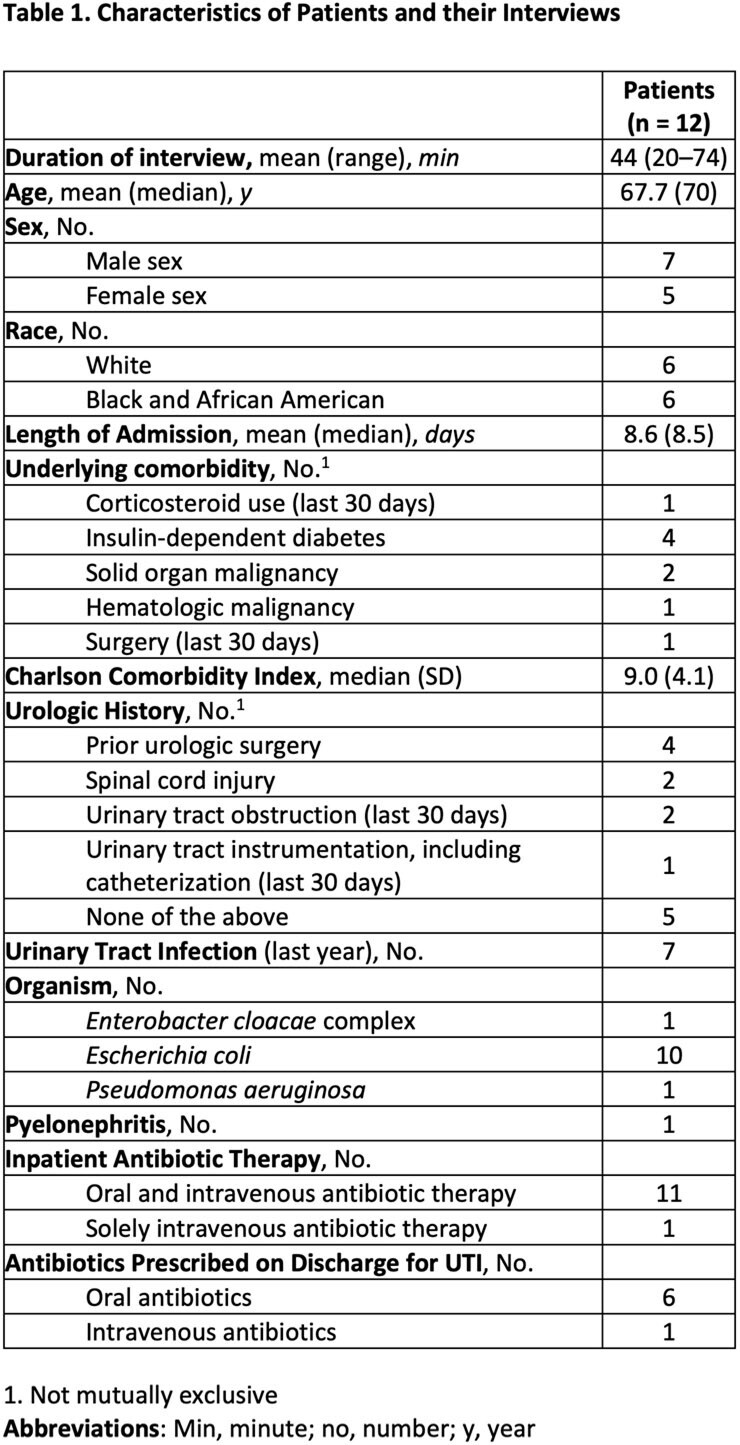

**Methods:**

Hospitalized patients with cUTI (defined by FDA guidance and determined by chart review) in an academic healthcare system in the Southeastern U.S. were interviewed 10-20 days after urine culture. Clinicians who cared for these patients for ≥2 days were eligible for interview, occurring 7-30 days after qualifying urine culture. Semi-structured, individual interviews were conducted between 10/2022–5/2023 by two interviewers via phone or Zoom. Interviews were informed by the Wilson and Cleary model of HRQoL and performed until saturation and information power were achieved. Rapid, matrix, and team-based qualitative techniques were used to facilitate analysis and presentation, summarizing within and across patient-clinician dyads (1 patient and 1 treating clinician) or triads (1 patient and 2 treating clinicians).

Table 2

**Figure:**
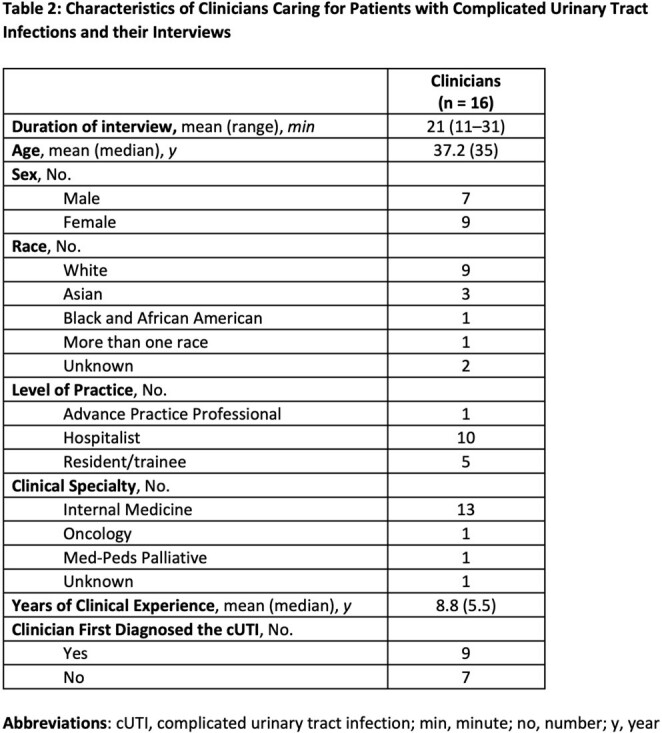

**Results:**

We interviewed 12 patients and 16 treating clinicians, comprising 8 patient-clinician dyads and 4 triads. Patient and clinician characteristics are reported in Tables 1 and 2. Our analysis identified four key concepts (Table 3): 1) The full range of impact of cUTI symptoms on patient functioning (e.g., emotional, cognitive functioning) was not consistently recognized by clinicians, 2) Clinicians’ perceptions of HRQoL were particularly influenced by infection characteristics, comorbidities, and other diagnoses, 3) Overall clinicians think HRQoL is important but approach their role in addressing HRQoL with varied experience and comfort, and 4) Different terms and level of detail were used by patients and clinicians to describe the impact of the cUTI.

Table 3

**Figure:**
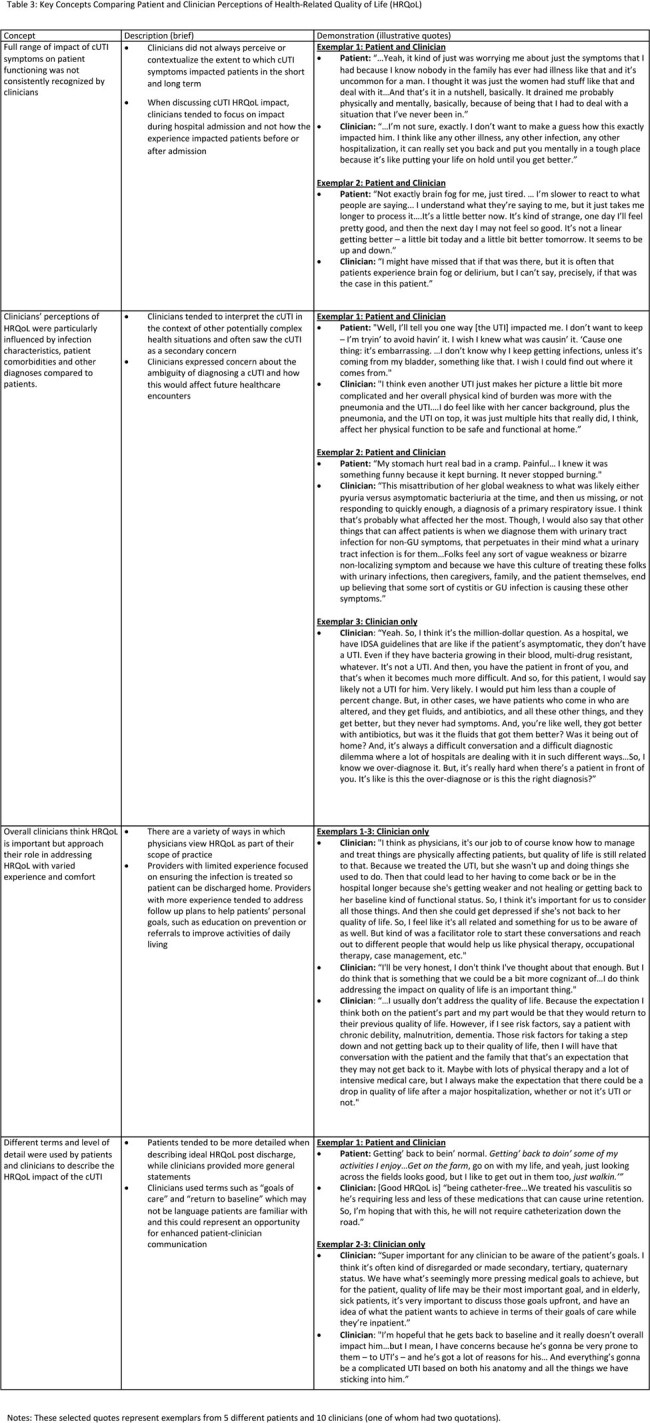

**Conclusion:**

While patients and clinicians both recognize that cUTIs impact HRQoL, there are important differences in how patients and clinicians perceive and describe this impact. Understanding how patient and clinician perceptions of HRQoL diverge can help improve design of patient-centered trials and identify areas for enhanced patient-clinician communication.

**Disclosures:**

**Engels N. Obi, PhD**, Merck & Co., Inc.: Employee|Merck & Co., Inc.: Stocks/Bonds (Public Company) **Alexandre H. Watanabe, PharmD**, Merck & Co., Inc.: Employee|Merck & Co., Inc.: Stocks/Bonds (Public Company) **Emre Yucel, PhD**, Merck: I am a full time Merck Employee and own stocks in the retirement plan provided by Merck.|Merck: Stocks/Bonds (Public Company) **Hayden Bosworth, PhD**, BeBetter therapeutics: Grant/Research Support|Boehringer Ingelheim: Advisor/Consultant|Boehringer Ingelheim: Grant/Research Support|Elton John Foundation: Grant/Research Support|Esperion: Grant/Research Support|Hilton foundation: Grant/Research Support|Improved Patient Outcomes: Grant/Research Support|Merck: Grant/Research Support|NHLBI: Grant/Research Support|Novo Nordisk: Grant/Research Support|Otsuka: Grant/Research Support|Pfizer: Grant/Research Support|Preventric Diagnostics: Board Member|sanofi: Advisor/Consultant|sanofi: Grant/Research Support|Veterans Health Administration: Grant/Research Support|Walmart: Advisor/Consultant **Helen Boucher, MD**, ASM: Honoraria|Elsevier: Honoraria|Sanford Guide: Honoraria **Deborah Collyar, B.Sci**, Apellis Pharmaceuticals, Inc.: Advisor/Consultant|Kinnate Biopharma: Advisor/Consultant **Sarah B. Doernberg, MD, MAS**, Basilea Pharmaceutica: Grant/Research Support|F2G Limited: Grant/Research Support|Genentech: Advisor/Consultant|Gilead Biosciences: Grant/Research Support|Janssen/J+J: Advisor/Consultant|Pfizer, Inc: Grant/Research Support|Regeneron, Inc: Grant/Research Support|Shinogi: Grant/Research Support **Vance G. Fowler, MD, MHS**, Affinergy: Advisor/Consultant|ArcBio: Stocks/Bonds (Private Company)|Armata: Advisor/Consultant|Astra Zeneca: Advisor/Consultant|Astra Zeneca: Grant/Research Support|Basilea: Advisor/Consultant|Basilea: Grant/Research Support|ContraFect: Advisor/Consultant|ContraFect: Grant/Research Support|Debiopharm: Advisor/Consultant|Destiny: Advisor/Consultant|EDE: Grant/Research Support|Genentech: Advisor/Consultant|Genentech: Grant/Research Support|GSK: Advisor/Consultant|Janssen: Advisor/Consultant|Karius: Grant/Research Support|MedImmune: Grant/Research Support|Merck: Grant/Research Support|sepsis diagnostics: Patent pending|UptoDate: Royalties|Valanbuio: Stocks/Bonds (Private Company)|Valanbuio: Stocks/Bonds (Private Company) **Heather King, PhD**, Merck Sharp & Dohme LLC, a subsidiary of Merck & Co., Inc., Rahway, NJ, USA.: Grant/Research Support

